# Long Non-coding RNA RP11-480I12.5 Promotes the Proliferation, Migration, and Invasion of Breast Cancer Cells Through the miR-490-3p-AURKA-Wnt/β-Catenin Axis

**DOI:** 10.3389/fonc.2020.00948

**Published:** 2020-07-07

**Authors:** Xinya Gao, Yuanhui Lai, Zhanqiang Zhang, Yanfei Ma, Zhizhai Luo, Yanghong Li, Ciqiu Yang, Guanming Lu, Jie Li

**Affiliations:** ^1^Department of Neurosurgery, First Affiliated Hospital of Sun Yat-sen University, Guangzhou, China; ^2^Department of Breast and Thyroid Surgery, Eastern Hospital of the First Affiliated Hospital of Sun Yat-sen University, Guangzhou, China; ^3^Department of Breast and Thyroid Surgery, First Affiliated Hospital of Sun Yat-sen University, Guangzhou, China; ^4^Department of Breast and Thyroid Surgery, Affiliated Hospital of Youjiang Medical University for Nationalities, Baise, China; ^5^Department of Breast and Thyroid Surgery, Guangdong General Hospital, Guangzhou, China

**Keywords:** breast cancer, RNA RP11-480I12.5, survival, proliferation, migration, invasion

## Abstract

**Background:** RP11-480I12. 5 is a newly identified long non-coding RNA (lncRNA) that has never been studied in breast cancer (BC). The biological function of RP11-480I12.5 in breast carcinoma and its underlying mechanism are still unknown.

**Methods:** We scanned The Cancer Genome Atlas (TCGA) database and identified RP11-480I12.5 as one of the most dysregulated lncRNAs. The level of RP11-480I12.5 was assessed in BC tissue samples and BC cell lines. The prognostic value of RP11-480I12.5 expression was assessed using the Kaplan–Meier method. The biological influence of RP11-480I12.5 on BC cell lines was studied using proliferation and Transwell migration and invasion assays.

**Results:** RP11-480I12.5 expression was upregulated in data from both the TCGA database and our own database. Moreover, Kaplan–Meier and Cox proportional hazard analyses indicated that high RP11-480I12.5 expression was related to poor overall survival. Moreover, RP11-480I12.5 promoted the proliferation, migration, and invasion of BC. RP11-480I12.5 promoted the expression of AURKA and the activation of the downstream Wnt/β-catenin pathway by sponging the microRNA (miRNA) miR-490-3p.

**Conclusion:** Taken together, our results indicate that RP11-480I12.5 is associated with tumor progression in BCs. Our findings indicate that the lncRNA RP11-480I12.5 promotes the proliferation, migration, and invasion of BC cells through the miR-490-3p-AURKA-Wnt/β-catenin axis, which may serve as a therapeutic target in the future.

## Background

Breast cancer (BC) is one of the most common cancers worldwide and the second leading cause of cancer-related mortality in women (1). Although advances in therapy have been made, patients still suffer from metastasis and recurrence (2–4). Immunotherapeutic strategies, such as chimeric antigen receptor (CAR) T cells and immune checkpoint inhibitors (such as PDL1 inhibitors), have been applied, but in most patients, chemotherapy and radiotherapy are still the most common treatments (5). Hence, more therapeutic targets are urgently needed.

Long non-coding RNAs (lncRNAs) are involved in many physiological and pathological processes in humans, such as cell division and differentiation. LncRNAs are reported to be involved in the progression of multiple kinds of carcinomas, such as gastric cancer (6) and lung cancers (7). Many studies have shown that lncRNAs contribute to multiple processes in the progression of breast carcinoma, such as proliferation, angiogenesis, and tissue invasion. The lncRNA HOXA-AS2 regulates the expression of SCN3A by sponging miR-106a (8), the lncRNA PTCSC3 inhibits triple-negative BC cell proliferation by downregulating lncRNA H19 expression (9), and LINC00511 knockdown enhances paclitaxel cytotoxicity in BC by regulating the miR-29c/CDK6 axis (10).

In the present study, we scanned The Cancer Genome Atlas (TCGA) database and identified RP11-480I12.5 as exhibiting significantly upregulated expression in BC. We next detected the expression difference between normal tissue and BC tissue. Then, we uncovered the biological function of RP11-480I12.5 through a series of experiments. We conclude that RP11-480I12.5 promotes the proliferation, migration and invasion of BC through the miR-490-3p-AURKA-Wnt/β-catenin axis.

## Methods

### Tissue Samples

All human tissue samples were obtained from the surgical suite at the First Affiliated Hospital of Sun Yat-Sen University and the Department of Breast and Thyroid Surgery, Affiliated Hospital of Youjiang Medical University for Nationalities after confirmation by a pathologist. The tissue specimens were obtained with the patients' written consent under a protocol approved by the institution's Institutional Review Board. Among the 101 patients enrolled, 21 had triple-negative disease, 23 had Her2+ disease, while the others had the luminal subtype.

### Quantitative Real-Time Polymerase Chain Reaction (qRT-PCR)

Total RNA was extracted using TRIzol reagent (Invitrogen, NY, USA) according to the manufacturer's instructions. qRT-PCR was performed using a SYBR green detection RT-PCR system (Takara, Japan). Actin was utilized for normalization. All samples were analyzed in triplicate. The 2^−ΔΔ*Ct*^ method was used to calculate the relative RNA expression. The probes are listed below:

Forward primer: ATTGCATTGCCAATTTGA

Reverse primer: GTCAAATAAAGTTTGGAAAAC.

### Cell Culture and a Cell Transfection Assay

The human mammary cancer cell lines MCF-10A, MDA-MB-453, BT-549, BT-474, MCF-7, and MDA-MB-231 were gifts from Professor Lv. Cells were cultured in Dulbecco's modified Eagle's medium (DMEM), minimum essential medium (MEM), or RPMI 1640 medium supplemented with 10% fetal bovine serum (FBS; Life Technologies, Grand Island, NY, USA), 1% penicillin G, and streptomycin.

### Cell Counting Kit-8 Assay

Cell Counting Kit-8 (CCK-8; Dojindo, Tabaru, Japan) was utilized to measure proliferation. Two hundred cells/well were seeded in 96-well-plates. The absorption values were measured at 24, 48, and 72 h after shRNA transfection. The experiments were repeated three times, and the data are shown as the mean ± standard deviation (SD).

### Colony Formation Assay

Transfected BC cells were seeded in a 6-well-plate at a density of 500 cells/well. After 2 weeks of culture, cell colonies were fixed with 4% paraformaldehyde and stained with 1% crystal violet. The colonies were examined and counted under a microscope.

### Transwell Migration and Invasion Assays

A Transwell system was applied to detect migration and invasion. For the invasion assay, Matrigel chambers (BD Biosciences, San Jose, CA, USA) were used to determine the effect of cells on invasion according to the manufacturer's instructions. A total of 5 × 10^4^ cells/well were resuspended in 250 μl of medium in the upper chamber (8-μm pore size, CoStar, Corning, NY, USA) of a Transwell system with no FBS, whereas the lower chamber was filled with 0.5 ml of medium supplemented with 10% FBS. After incubating for 24 h at 37°C, the invasive cells were fixed with 100% methanol and stained with 0.5% crystal violet before counting under an inverted microscope. For the migration assay, the indicated cells were plated in uncoated upper chambers. The number of transmembrane cells was estimated under a microscope (Nikon, Tokyo, Japan) at 200 × magnification.

### Lentiviral Production and Stable Cell Line Construction

Lentiviral vectors expressing shRNA and RP11-480I12.5 were cotransfected with the packaging vectors psPAX2 and pMD2G (Addgene) into HEK293FT cells using Lipofectamine 3000 to produce lentiviruses in accordance with the manufacturer's instructions. To establish stable cell lines, cells were transduced by using the above lentiviruses with polybrene (8 mg/ml, Sigma). After incubating for 72 h, the cells were selected with 2 mg/ml puromycin for 3 days.

### Statistical Analysis

All data analyses were performed with SPSS 20.0 statistical software. The χ^2^-test was used to analyze the relationships between categorical variables. Differences between groups were compared by Student's *t*-test. Cox regression and Kaplan–Meier methods were used to analyze overall survival (OS), and the cutoff value of RP11-480I12.5 in patients was the mean of the whole cohort in both the TCGA database and our in-house database. *p* < 0.05 was considered to indicate significant difference from the control.

## Results

### RP11-480I12.5 Expression Is Upregulated in BC Tissue and Associated With a Poor Prognosis

**Figure 1:**
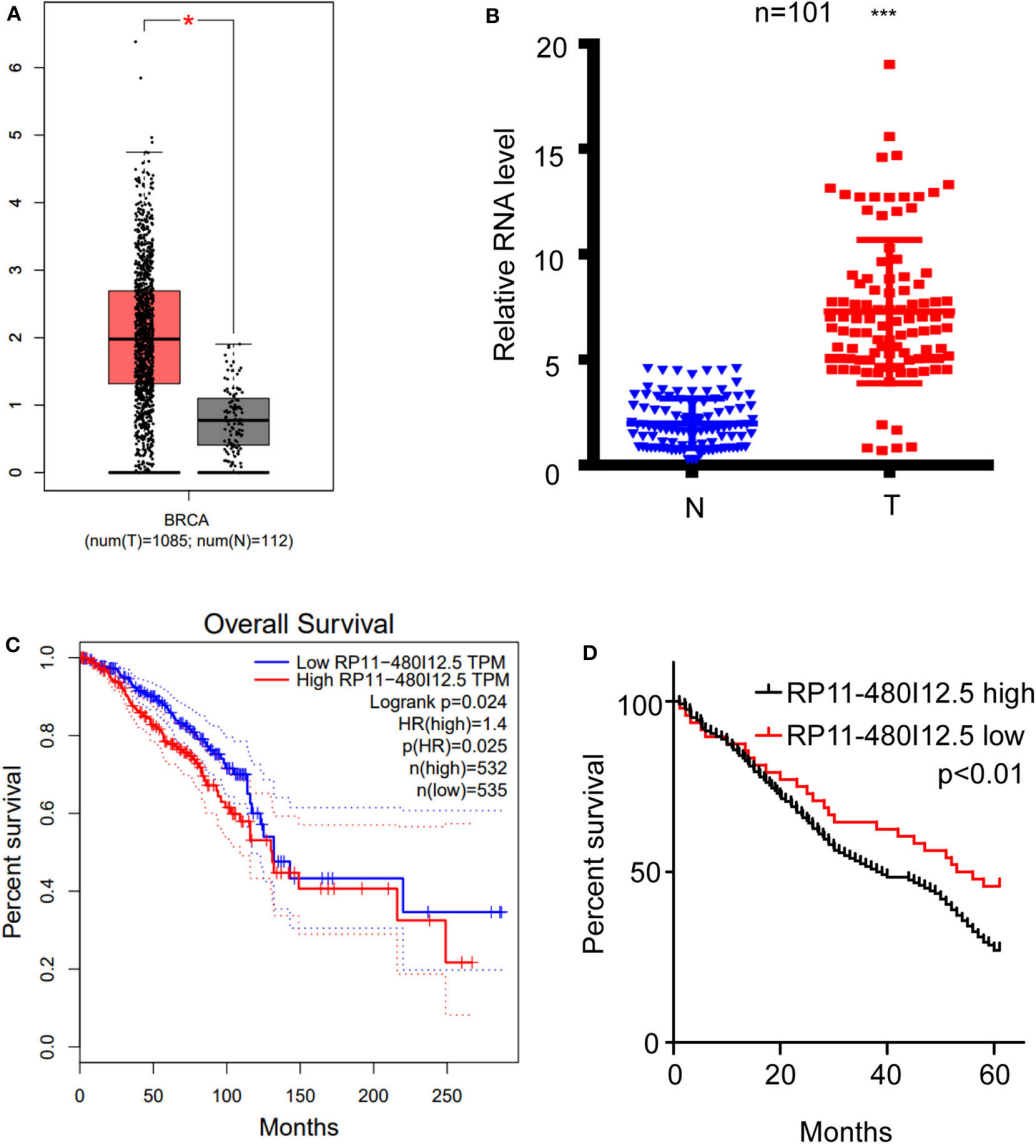
RP11-480I12.5 was upregulated in BC and was negatively correlated with prognosis. **(A)** The relative RNA level of RP11-480I12.5 in TCGA database (*p < 0.05). **(B)** The relative RNA level of RP11-480I12.5 in our in-house database (***p < 0.001). **(C)** Survival analysis of patients in TCGA database (Log rank test *p* < 0.05). **(D)** Survival analysis of patients in our in-house database (Log rank test *p* < 0.05).

We scanned the TCGA database and identified RP11-480I12.5 as one of the lncRNAs contributing to the progression of BC (Figure 1A, *p* < 0.05). To further verify this hypothesis, we analyzed the expression differences between 50 paired normal tissue samples and tumor samples with a qRT-PCR assay. RP11-480I12.5 expression was significantly upregulated in the tumor tissue specimens (*p* < 0.001) (Figure 1B, Figure S2). Moreover, we analyzed the correlation between RP11-480I12.5 and the OS of patients. The results for both the TCGA database (*p* = 0.025) (Figure 1C) and our own database showed that RP11-480I12.5 expression was negatively associated with prognosis (*p* < 0.05) (Figure 1D).

### RP11-480I12.5 Promotes the Proliferation of BC Cells

**Figure 2:**
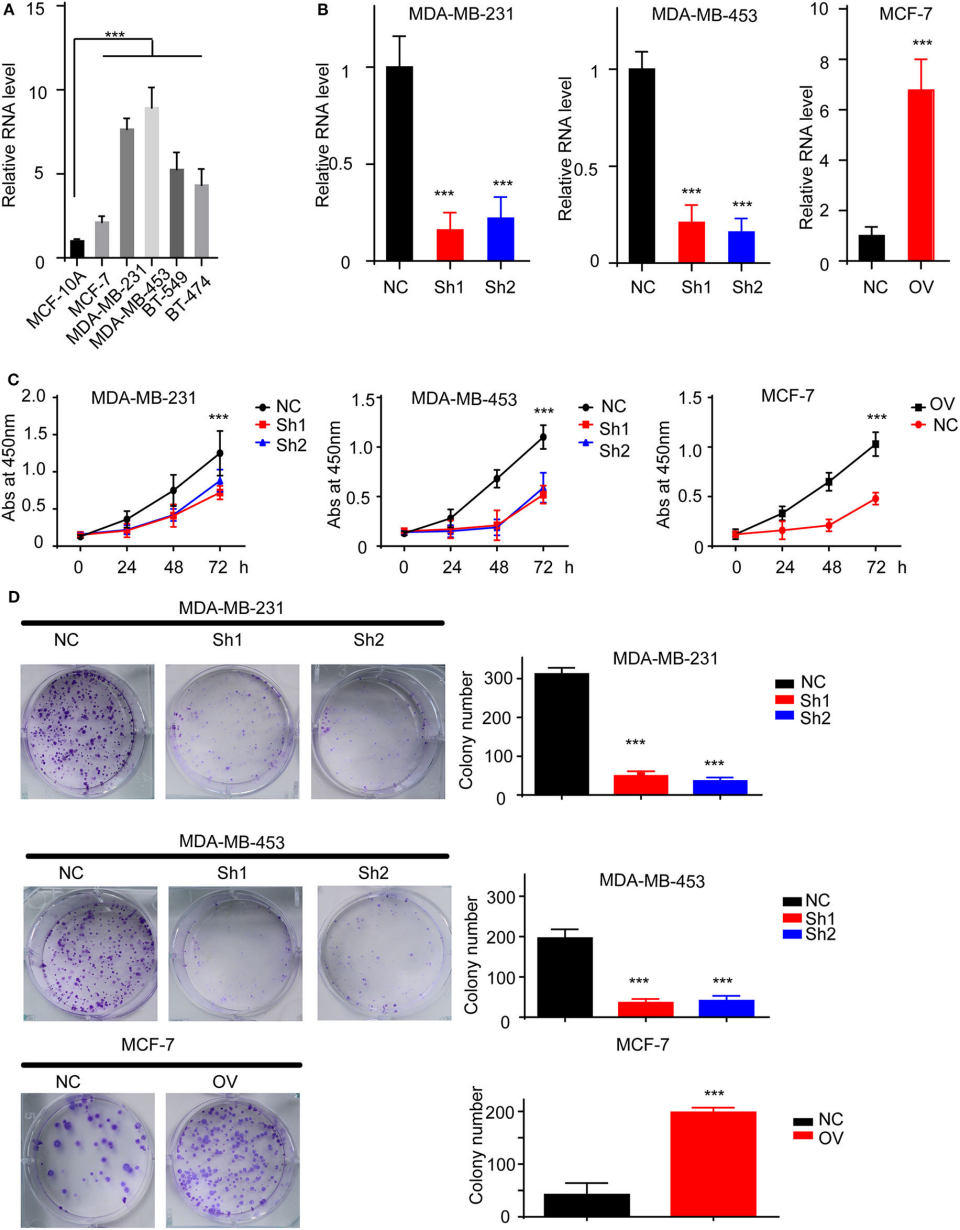
RP11-480I12.5 promotes the proliferation of BC. **(A)** The relative level of RP11-480I12.5 in BC cell lines (***p < 0.001). **(B)** The relative level of RP11-480I12.5 of stable cell line (***p < 0.001). **(C)** The abs at 450 nm of different cell lines in the CCK-8 assay (***p < 0.001). **(D)** Left: The representative image of colony formation assay. Right: The statistical analysis of colony formation assay (***p < 0.001).

We have suggested that RP11-480I12.5 expression is upregulated in BC and negatively associated with prognosis. To further uncover the biological function of RP11-480I12.5, we detected the expression pattern in BC cell lines. A BC cell line harbored a higher level of RP11-480I12.5 than the control normal cell line MCF-10A (*p* < 0.001) (Figure 2A). We next established RP11-480I12.5 stable-knockdown cell lines with the MDA-MB-231 and MDA-MB-453 cell lines and an RP11-480I12.5-overexpressing cell line with the MCF-7 cell line. The relative RNA levels are shown in Figure 2B (*p* < 0.001). We performed a series of experiments measuring the proliferative abilities of the cell lines described above. The MDA-MB-231, MDA-MB-453, and MCF-7 cells with higher RP11-480I12.5 expression proliferated faster (Figures 2C,D, *p* < 0.001). RP11-480I12.5 promotes proliferation in short- and long-term manners.

### RP11-480I12.5 Promotes the Migration and Invasion of BC

**Figure 3:**
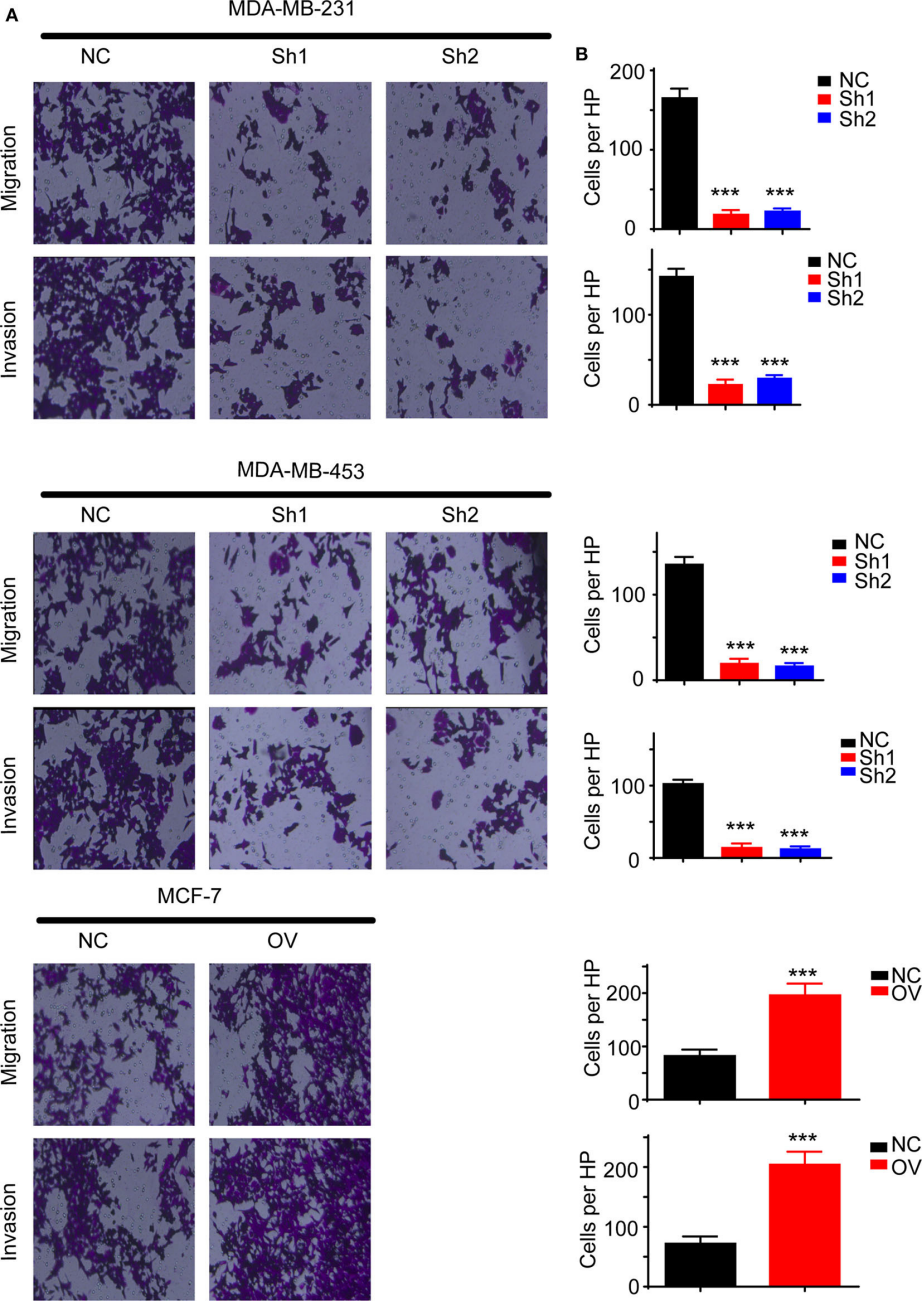
RP11-480I12.5 promotes the migration and invasion of BC. **(A)** The representative image of Transwell and invasion chambers. Cells were seeded into the Transwell and invasion chambers; after incubation, cells were stained with crystal violet. **(B)** The statistical analysis of Transwell and invasion chamber (***p < 0.001); the cell numbers of each HP were calculated to measure the migration and invasion ability.

**Figure 4:**
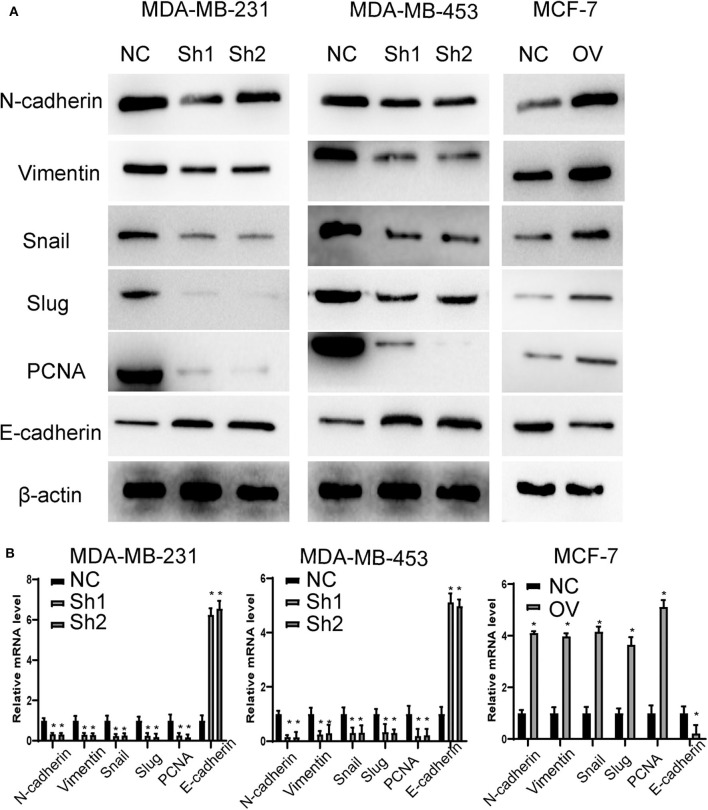
RP11-480I12.5 promotes the EMT in BC. **(A)** The Western blot of EMT marker and PCNA in BC. EMT markers and PCNA were the most common markers reflecting migration and invasion and proliferation. We detected the protein level of EMT markers and PCNA to measure the migration, invasion, and proliferation ability. **(B)** The relative mRNA level of EMT marker and PCNA in BC (*p < 0.05).

Recurrence and metastasis are the most common factors contributing to patient death. We next analyzed the migratory and invasive abilities of the cell lines described above. The results showed that cells with higher levels of RP11-480I12.5 developed improved migratory and invasive abilities. A Transwell assay indicated that cells with a higher level of RP11-480I12.5 migrated more easily and moved through the semipermeable membrane more easily. In the invasion chamber assay, cells with overexpression degraded the matrix gel, invaded into the membrane, and migrated through the membrane more easily than the control cell lines (*p* < 0.001) (Figures 3A,B). Epithelial–mesenchymal transition (EMT) is the most common change in cancer cells during migration and invasion. EMT markers are the most common markers reflecting the mesenchymal status of cancer cells. We previously showed that RP11-480I12.5 promoted the migration and invasion of BC cells. We next detected EMT markers and the proliferation marker PCNA. The results showed that cells with a higher level of RP11-480I12.5 tended to transition from epithelial cells to mesenchymal cells. The expression of mesenchymal markers, such as N-cadherin, vimentin, snail, and slug, and PCNA increased, while that of E-cadherin decreased at both the RNA and protein levels (Figures 4A,B, *p* < 0.05, Figure S1A).

### RP11-480I12.5 Promotes the Expression of AURKA and the Activation of the Downstream Wnt/β-Catenin Pathway

**Figure 5:**
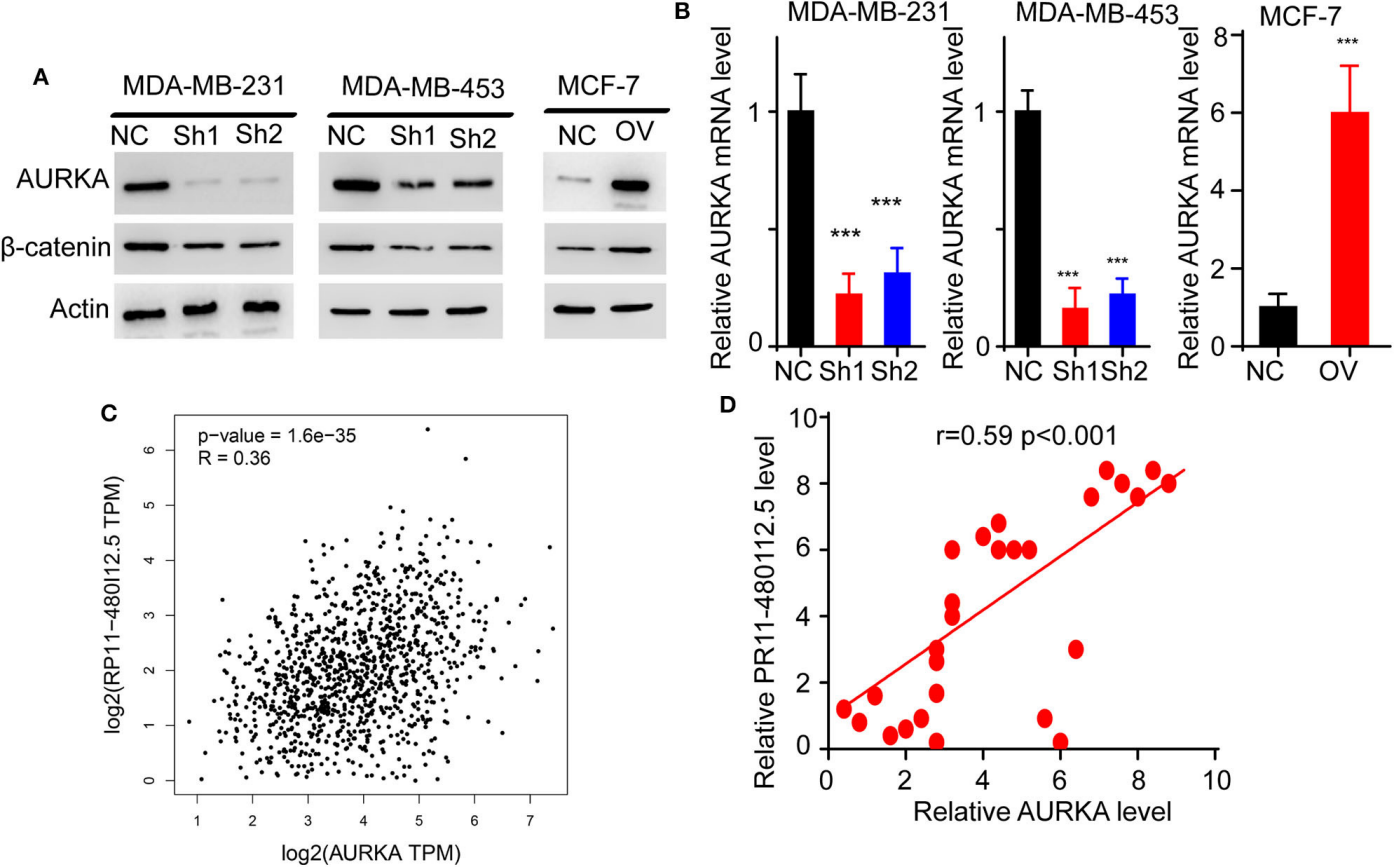
RP11-480I12.5 promotes the expression of AURKA. **(A)** Western blot of AURKA of different cell lines. **(B)** Relative mRNA level of AURKA of different cell lines (***p < 0.001). **(C)** Regression analysis of RP11-480I12.5 and AURKA in TCGA database. The correlation between AURKA and RP11-480I12.5 in TCGA database was measured to further confirm the relation between AURKA and RP11-480I12.5. **(D)** Regression analysis of RP11-480I12.5 and AURKA in our own database.

To uncover the mechanism underlying the role of RP11-480I12.5 in BC, we detected the expression of AURKA in RP11-480I12.5-knockdown and RP11-480I12.5-overexpressing cell lines and found that the expression of AURKA at both the protein and mRNA levels was increased in the cells with a higher level of RP11-480I12.5 and decreased in the cells with a lower level of RP11-480I12.5. The level of the downstream molecule β-catenin correlated with the level of AURKA (Figures 5A,B, *p* < 0.001, Figure S1B). We next examined the correlation between RP11-480I12.5 and AURKA in the TCGA database and our own database; RP11-480I12.5 expression was positively correlated with the level of AURKA in the data from both the TCGA database (Figure 5C, *R* = 0.36, *p* < 0.001) and our in-house database (Figure 5D, *R* = 0.59, *p* < 0.001). The results indicated that RP11-480I12.5 may exert its function through AURKA and the downstream Wnt/β-catenin pathway.

### RP11-480I12.5 Promotes Proliferation, Migration, Invasion, and EMT in an AURKA-Dependent Manner

**Figure 6:**
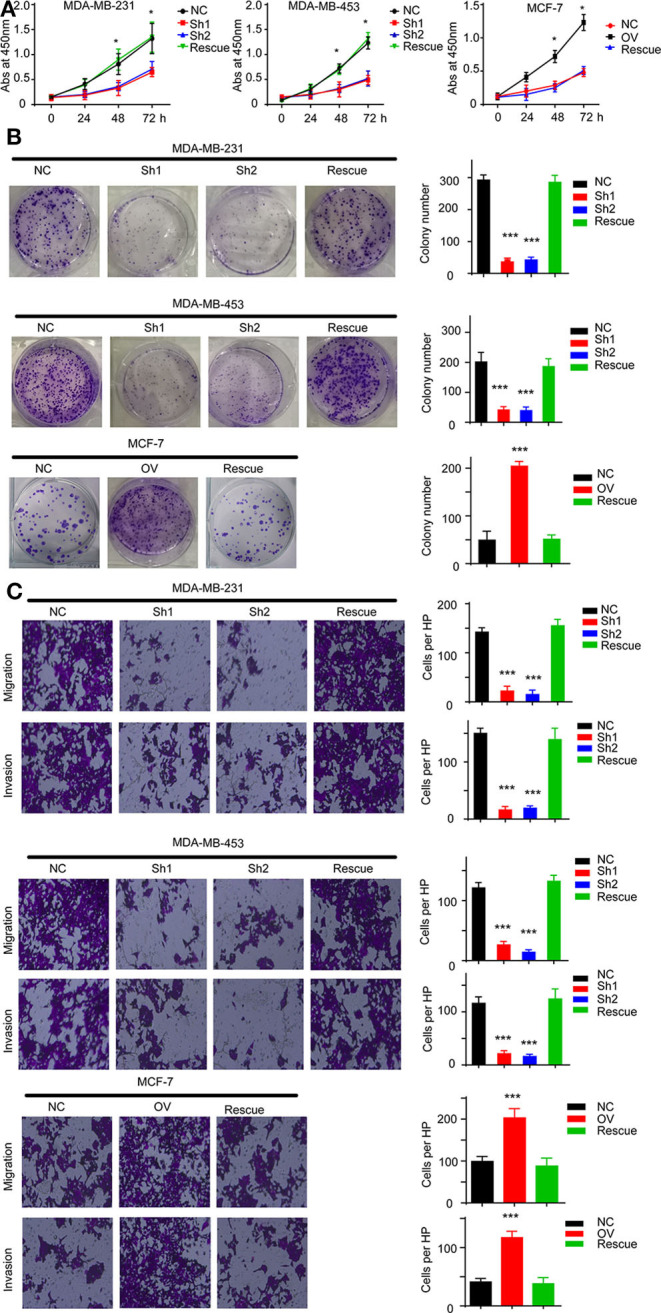
RP11-480I12.5 exerts its function in an AURKA-dependent manner. **(A)** The abs at 450 nm of different cell lines in CCK-8 assay (*p < 0.05). **(B)** Left: The representative image of colony formation assay. Right: The statistical analysis of colony formation assay (***p < 0.001). **(C)** Left: The representative image of Transwell and invasion chamber. Right: The statistical analysis of Transwell and invasion chamber (***p < 0.001).

**Figure 7:**
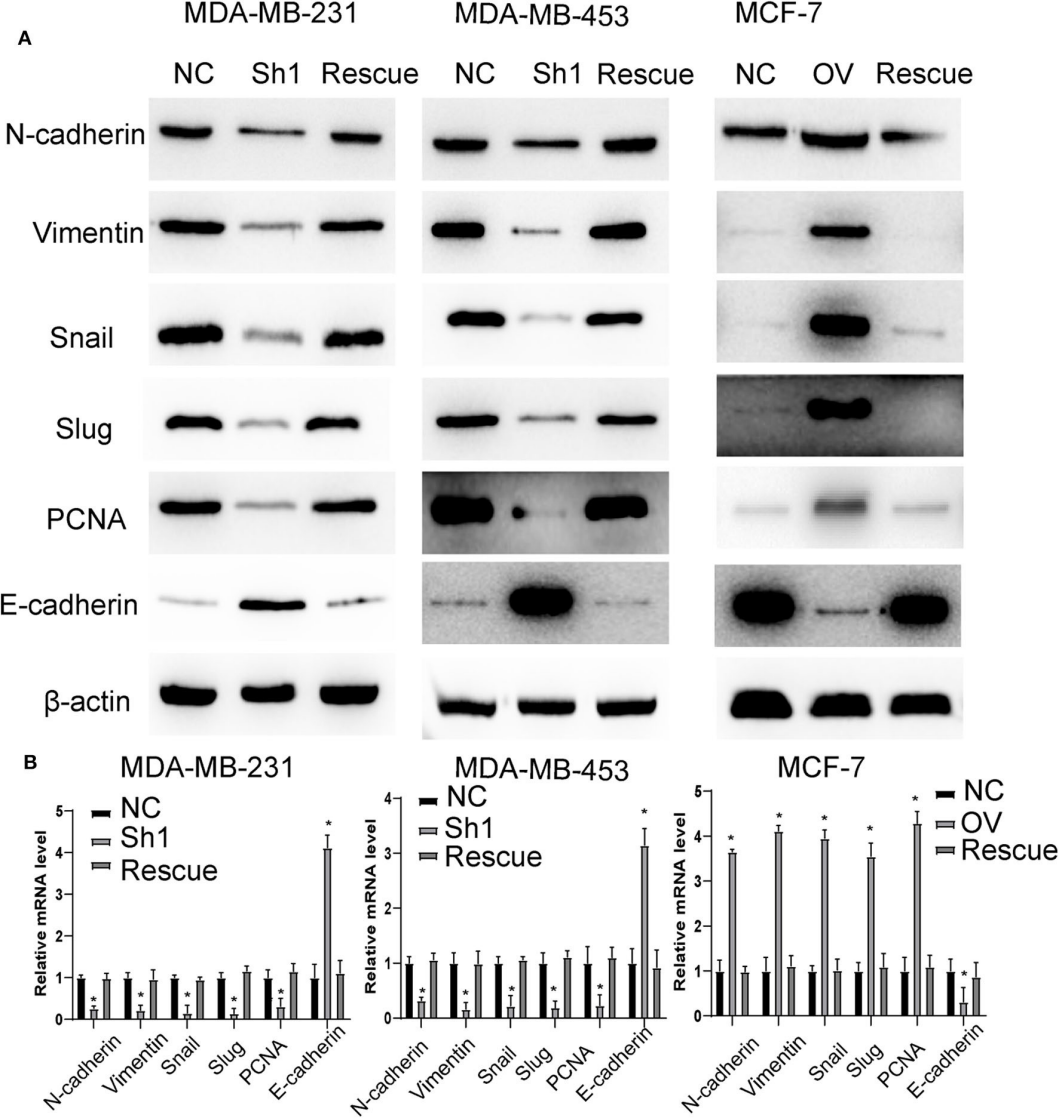
RP11-480I12.5 promotes EMT in an AURKA-dependent manner. **(A)** The Western blot of EMT marker and PCNA in BC. **(B)** The relative mRNA level of EMT marker and PCNA in BC (*p < 0.05).

For further verification, we established a rescue cell line, where we re-expressed AURKA in an RP11-480I12.5-knockdown cell line and knocked down AURKA expression in an RP11-480I12.5-overexpressing cell line. We next performed a series of experiments and found that proliferation and invasion were restored in the AURKA re-expressing cell line and abolished in the AURKA-knockdown cell line (Figures 6A–C, *p* < 0.001), indicating that RP11-480I12.5 exerts its function in an AURKA-dependent manner. We next detected EMT markers and PCNA, and the results showed that the EMT markers were completely restored at both the RNA and protein levels in the rescue cell line (Figures 7A,B, *p* < 0.05, Figure S1C), indicating that RP11-480I12.5 promotes EMT in an AURKA-dependent manner. Thus, we have proven that RP11-480I12.5 promotes proliferation, migration, and invasion through AURKA and the downstream Wnt/β-catenin pathway.

### RP11-480I12.5 Promotes the Expression of AURKA by Inhibiting miRNA miR-490-3p-Mediated AURKA Degradation

**Figure 8:**
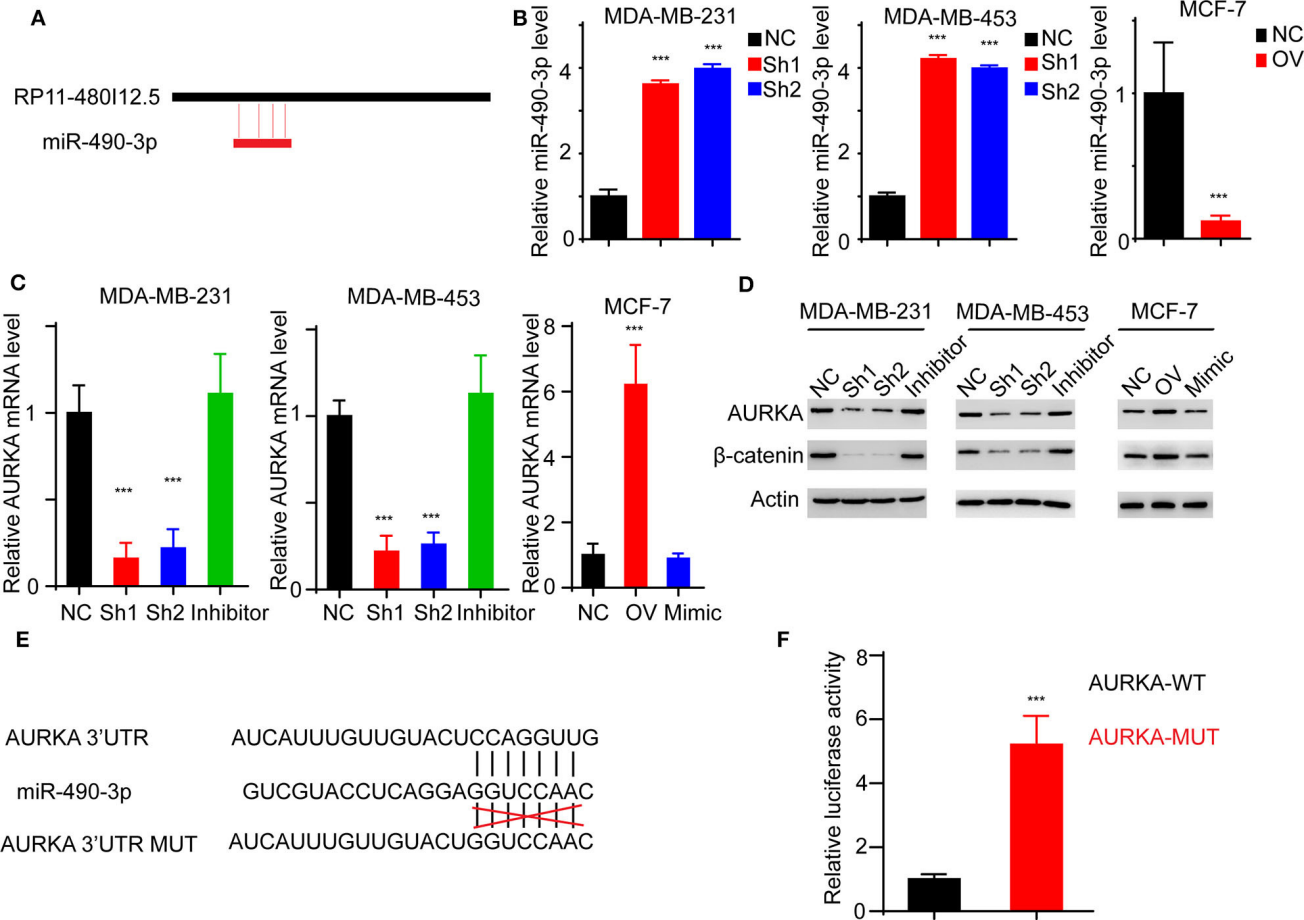
RP11-480I12.5 promotes the expression of AURKA through inhibiting miR-490-3p-mediated AURKA degradation. **(A)** The schematic model of miR-490-3p binding to RP11-480I12.5. **(B)** The relative miR-490-3p level in different cell lines. We detected the miR-490-3p level and found that miR-490-3p was negatively correlated with RP11-480I12.5. **(C)** The relative mRNA level of AURKA of different cell lines (***p < 0.001). To further confirm the sponge effect, we applied established cell lines and detected the mRNA level of AURKA. **(D)** Western blot of AURKA and β-catenin. **(E)** The schematic model of miR-490-3p binding to AURKA premRNA. **(F)** The relative luciferase activity in cells transfected with AURKA-WT and AURKA-MUT (***p < 0.001).

**Figure 9:**
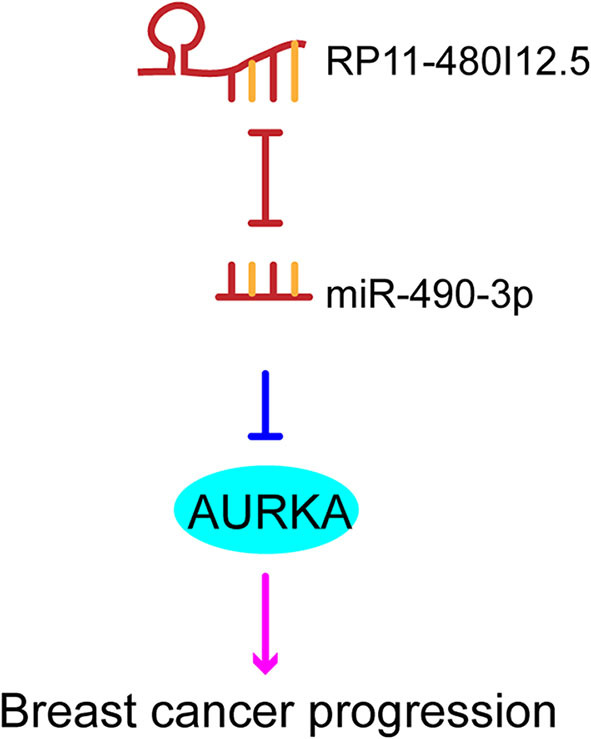
The schematic model of RP11-480I12.5, miR-490-3p, and AURKA.

We have proven that RP11-480I12.5 exerts its function through AURKA and the downstream Wnt/β-catenin pathway. We hypothesized that RP11-480I12.5 sponges microRNAs (miRNAs) that target the mRNA of AURKA. We further predicted the potential binding miRNAs by using http://mirdb.org/miRDB/index.html and http://www.mircode.org/index.php, and we selected miR-490-3p as the potential target miRNA (Figure 8A). To verify this hypothesis, we detected the miR-490-3p level in the RP11-480I12.5-knockdown and RP11-480I12.5-overexpressing cell lines. Cells with a higher level of RP11-480I12.5 had lower levels of miR-490-3p (Figure 8B, *p* < 0.001). We next transfected a miR-490-3p inhibitor into the RP11-480I12.5-knockdown cell line and a miR-490-3p mimic into the RP11-480I12.5-overexpressing cell line. We then detected the expression level of AURKA and found that the level of AURKA in the rescue cell line was completely restored, indicating that RP11-480I12.5 exerts its function through miR-490-3p (Figures 8C,D, *p* < 0.001, Figure S1D). MiRNAs are well-known non-coding RNAs engaged in the posttranscriptional regulation of gene expression. We next predicted the binding site of miRNA-490-3p in the AURKA pre-mRNA and found that miR-490-3p could bind to the 3′-untranslated region (UTR) of the pre-mRNA of AURKA (Figure 8E). We constructed a dual-luciferase reporter system, transfected wild-type AURKA (AURKA WT) or AURKA with a 3′-UTR mutation into the HEK293 cell line, and examined the relative luciferase activity. The results showed that the luciferase activity of the AURKA mutant was significantly increased compared with that of AURKA WT, indicating that miR-490-3p directly binds to the 3′-UTR of AURKA and promotes its degradation (Figure 8F, *p* < 0.001). Thus, we demonstrate that the lncRNA RP11-480I12.5 promotes the proliferation, migration, and invasion of BC cells through the miR-490-3p-AURKA-Wnt/β-catenin axis (Figure 9).

## Discussion

Breast carcinoma is the most common malignancy in women worldwide. Although many advancements have been made, many patients still suffer from recurrence and metastasis. Recent studies have shown that many lncRNAs participate in the tumorigenesis and progression of BC. Liu et al. showed that the lncRNA HOTAIR promotes the invasion of BC cells through the chondroitin sulfotransferase CHST15 (11). Zhang revealed the oncogenic role of NAMPT-AS in driving BC metastatic progression in triple-negative BC (12). Keshavarz, M and Asadi, M H found that the lncRNA ES1 controlled the proliferation of BC cells by regulating the Oct4/Sox2/miR-302 axis. Our study identified a new lncRNA, RP11-480I12.5, that had not been studied previously in BC and found that RP11-480I12.5 could promote proliferation, migration, and invasion, indicating that this lncRNA may be a therapeutic target.

MiR-490-3p is a well-studied miRNA that is involved in the progression of tumorigenesis. MiRNA-mediated posttranscriptional regulation is one of the most important types of epigenetic regulation. MiR-490-3p can be sponged by many kinds of lncRNAs and targets multiple pre-mRNAs. Mu et al. showed that the lncRNA CCAT1 upregulates TGFbetaR1 expression by sponging miR-490-3p to promote TGFbeta1-induced EMT in ovarian cancer cells (13). The lncRNA TP73-AS1 suppresses triple-negative BC cell vasculogenic mimicry by targeting the miR-490-3p/TWIST1 axis (14). CircSLC3A2 functions as an oncogenic factor in hepatocellular carcinoma by sponging miR-490-3p and regulating PPM1F expression (15). However, the role of miR-490-3p has rarely been studied. Zhang showed that miR-490-3p inhibited growth and invasion in triple-negative BC, but the underlying mechanism is still unknown. Our study showed that the lncRNA RP11-480I12.5 could sponge miR-490-3p, which inhibited its function.

The AURKA kinase is a serine/threonine kinase that is distributed in many kinds of cellular organisms and is essential for mitosis and cytokinesis. AURKA is reported to be involved in the progression of many kinds of cancers, such as gastric cancer and colon cancers (16, 17). AURKA has been reported to promote cell proliferation through AKT and the Wnt/β-catenin pathway (18). AURKA was also reported to contribute to the stemness (19) and chemoresistance (20) in BC cells. Combination of targeting AURKA and p21 was recently reported to be efficient for BC (21). The Wnt/β-catenin pathway is important in maintaining stemness and is overactivated in many cancers.

In the present research, we found that RP11-480I12.5 was commonly overexpressed in BC. RP11-480I12.5 promoted the expression of AURKA, which could be totally abolished with a miR-490-3p mimic. Thus, we conclude that the lncRNA RP11-480I12.5 promotes the proliferation, migration, and invasion of BC cells through the miR-490-3p-AURKA-Wnt/β-catenin axis.

## Conclusion

Taken together, our results indicate that RP11-480I12.5 is associated with tumor progression in BCs. Our findings indicate that the lncRNA RP11-480I12.5 promotes the proliferation, migration, and invasion of BC cells through the miR-490-3p-AURKA-Wnt/β-catenin axis, which may serve as a therapeutic target in the future.

## Data Availability Statement

All datasets generated for this study are included in the article/Supplementary Material.

## Ethics Statement

The studies involving human participants were reviewed and approved by the ethics committee of Affiliated Hospital of Youjiang Medical University for Nationalities, the ethics committee of Eastern Hospital of the First Affiliated Hospital of Sun Yat-sen University, the ethics committee of the First Affiliated Hospital of Sun Yat-sen and the ethics committee of Guangdong general hospital with the 1964 helsinki declaration and its later amendments or comparable ethical standards. The patients provided written informed consent to participate in this study.

## Author Contributions

JL, GL, and CY designed the research. All authors were engaged into the performance of experiments and data analysis. All authors contributed to the article and approved the submitted version.

## Conflict of Interest

The authors declare that the research was conducted in the absence of any commercial or financial relationships that could be construed as a potential conflict of interest.

## Supplementary Material

Figure S1 The relative densitometry of western blot. **(A)** The relative densitometry of western blot in Figure 4 (^***^p < 0.001). **(B)** The relative densitometry of western blot in Figure 5 (^***^p < 0.001). **(C)** The relative densitometry of western blot in Figure 7 (^***^p < 0.001). **(D)** The relative densitometry of western blot in Figure 8 (^***^p < 0.001).

Figure S2 The relative RNA level in different subtypes of breast cancers. All the patients were divided into 3 subtypes according to the pathology results. The RNA level in different subtypes was detected.
