# Supplementary figures and images for: Long Non-coding RNA RP11-480I12.5 Promotes the Proliferation, Migration, and Invasion of Breast Cancer Cells Through the miR-490-3p-AURKA-Wnt/β-Catenin Axis
Source: Front Oncol. 2020 Jul 7;10:948. doi: 10.3389/fonc.2020.00948 (PMC7358571; doi:10.3389/fonc.2020.00948)

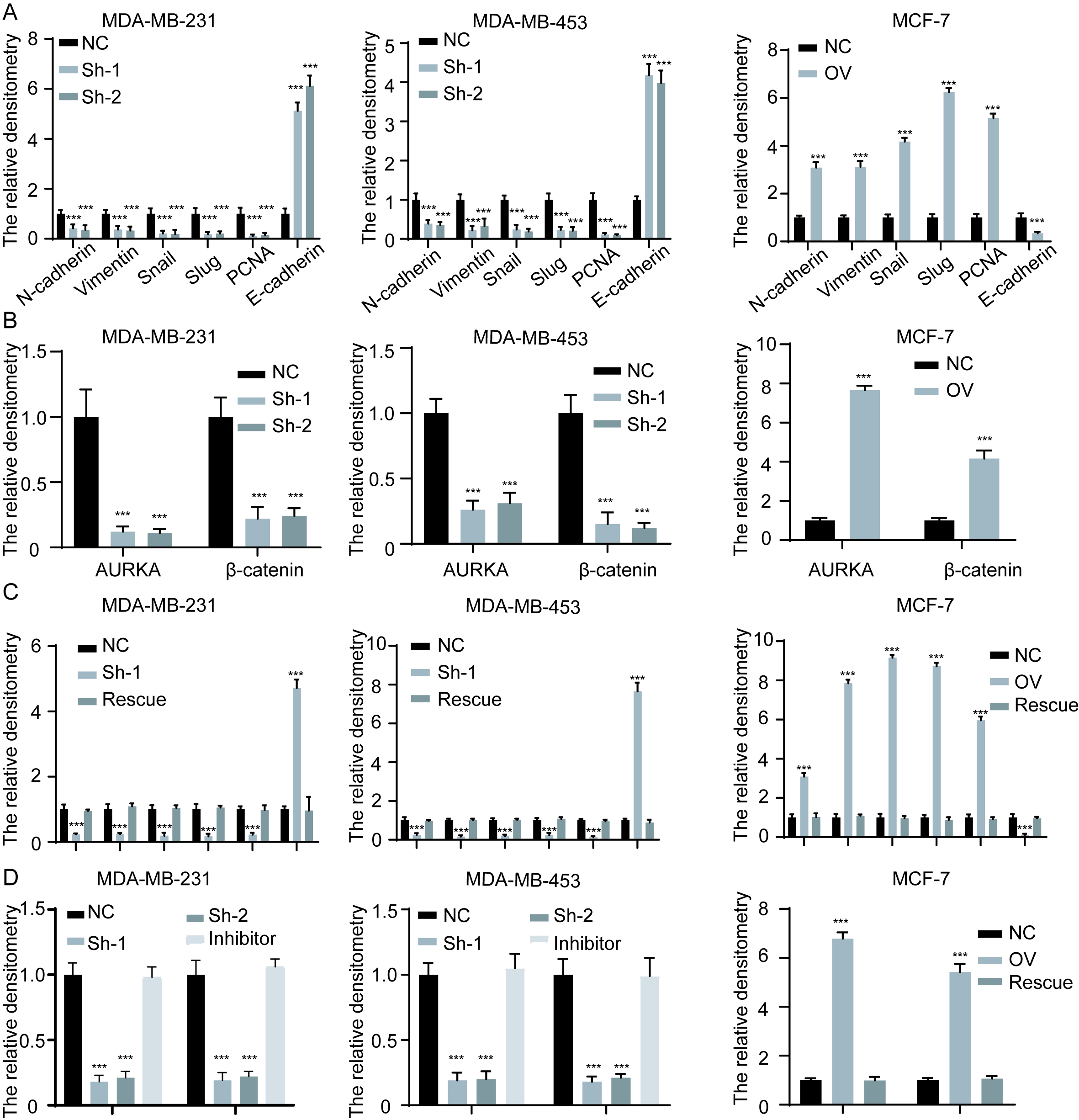

Supplement: Figure S1 — The relative densitometry of western blot. (A) The relative densitometry of western blot in Figure 4 (***p < 0.001). (B) The relative densitometry of western blot in Figure 5 (***p < 0.001). (C) The relative densitometry of western blot in Figure 7 (***p < 0.001). (D) The relative densitometry of western blot in Figure 8 (***p < 0.001). [file Image_1.tif]

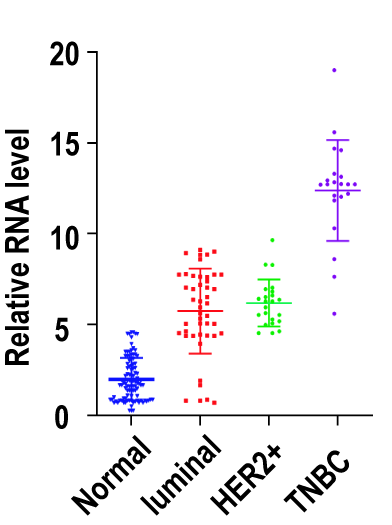

Supplement: Figure S2 — The relative RNA level in different subtypes of breast cancers. All the patients were divided into 3 subtypes according to the pathology results. The RNA level in different subtypes was detected. [file Image_2.tif]
